# FDG-PET/CT versus bone marrow biopsy in bone marrow involvement in newly diagnosed paediatric lymphoma: a systematic review and meta-analysis

**DOI:** 10.1186/s13018-021-02521-3

**Published:** 2021-08-09

**Authors:** Zhizhuo Li, Chengxin Li, Bingrong Chen, Lijun Shi, Fuqiang Gao, Peixu Wang, Wei Sun

**Affiliations:** 1 Department of Orthopedics, Peking University China-Japan Friendship School of Clinical Medicine, 2 Yinghuadong Road, Chaoyang District, Beijing, 100029 China; 2 Department of Orthopedics, Graduate School of Peking Union Medical College, China-Japan Friendship institute of Clinical Medicine, 2 Yinghuadong Road, Chaoyang District, Beijing, 100029 China; 3 Beijing Key Laboratory of Immune Inflammatory Disease, China-Japan Friendship Hospital, Peking Union Medical College, 2 Yinghuadong Road, Chaoyang District, Beijing, 100029 China

**Keywords:** Paediatric lymphoma, Bone marrow involvement, FDG-PET/CT, Bone marrow biopsy, Meta-analysis

## Abstract

**Background:**

Bone marrow infiltration (BMI) is a devastating stage of paediatric lymphoma. Prompt diagnosis of BMI in newly diagnosed paediatric lymphoma patients is critical but can be very challenging at present.

**Methods:**

We systematically retrieved studies from PubMed, EMBASE, and the Cochrane Library. Data extraction and quality assessment were performed by two reviewers independently. A total of nine eligible studies were included in the quantitative analysis.

**Results:**

The pooled sensitivity and specificity of FDG-PET/CT for diagnosing BMI in newly diagnosed paediatric lymphoma patients were 0.97 (95% confidence interval [CI], 0.93 to 0.99) and 0.99 (95% CI, 0.98 to 0.99), respectively. The pooled PLR, NLR, and DOR were 79.9 (95% CI, 42.7 to 149.6), 0.03 (95% CI, 0.01 to 0.17), and 2414.6 (95% CI, 989.6 to 5891.4), respectively. The AUC of FDG-PET/CT for BMI was 1.00 (95% CI, 0.99 to 1.00). Compared with FDG-PET/CT, BMB had a lower pooled sensitivity (0.44, 95% CI, 0.34 to 0.55) and comparable pooled specificity (1.00, 95% CI, 0.92 to 1.00).

**Conclusion:**

Compared with BMB, FDG-PET/CT was a more valuable diagnostic method for evaluating BMI in paediatric Hodgkin and non-Hodgkin lymphoma patients with extremely high diagnostic accuracy.

## Introduction

Lymphoma is one of the most common paediatric malignancies, with a prevalence rate of 12–15%, following acute leukaemia and malignant brain tumours [1]. The detection of bone marrow involvement (BMI) in lymphoma is important for accurate staging and management of the disease because the presence of BMI indicates the highest stage of lymphoma (Ann Arbor stage IV), which influences both treatment and prognosis [2]. The gold standard procedure for evaluating BMI is bone marrow biopsy (BMB) [3]. However, BMB explores only a limited part of the bone marrow, generally the unilateral or bilateral iliac crest, so focal bone marrow involvement may be missed. In addition, as an invasive procedure, BMB can cause pain to patients [4]. Despite these defects, BMB has been routinely used to assess BMI for many years.

As a glucose analogue, F-18 FDG provides information about glucose metabolism in normal and abnormal tissues, particularly in FDG-avid malignancies. F-18 FDG positron emission tomography/computed tomography (FDG-PET/CT) can simultaneously assess the structural anatomy and metabolic activity level of a tumour, which may be very useful for detecting BMI in paediatric lymphoma patients and may eliminate the need for BMB [5]. Because lymphoma is almost always FDG-avid, FDG-PET/CT has been widely used to stage newly diagnosed lymphoma, including the detection of BMI, and it has been reported to have high sensitivity and accuracy [6].

The clinical value of FDG-PET/CT in assessing paediatric lymphoma BMI is still under debate and investigation. In recent years, several studies have been published on the application of FDG-PET/CT for detecting BMI in paediatric lymphoma patients. However, because of the heterogeneity of study quality and low incidence of BMI in these patients [1], the results of these studies are inconclusive. The aim of this systematic review and meta-analysis was to synthesize published data on the accuracy of FDG-PET/CT in detecting BMI in newly diagnosed paediatric lymphoma patients and to determine whether BMB is still necessary for these patients.

## Material and methods

The methodological approach to evidence searching and synthesis described in this article was based on the Cochrane Collaboration’s diagnostic test accuracy method [7]. We performed the current systematic review in accordance with the standards of the Preferred Reporting Items for Systematic Reviews and Meta-Analyses (PRISMA) in reporting the findings of this review [8]. No ethical approval or informed consent was required for this article because all data were retrieved from published literature. Searching for studies, identifying eligibility, extracting data, and assessing quality were performed by two investigators independently. Any disagreement was resolved through discussion, and the two researchers had to come to a consensus.

### Search strategy

Three electronic databases, PubMed, EMBASE, and Cochrane Library, were searched for entries recorded from the time of database inception to March 10, 2021. Vocabulary and syntax were specifically adapted according to the database. We used “bone marrow infiltration” or “bone marrow involvement” as our diagnosis of interest and “positron emission tomography” or “PET” or “positron emission tomography/computed tomography” or “PET/CT” as our target index. The following group terms were used for searching: ((((child or teen or adolescent or paediatric or infant or newborn)) AND (“positron emission tomography” or “PET” or “positron emission tomography/computed tomography” or “PET/CT”)) AND (“bone marrow infiltration” or “bone marrow involvement”)) AND (Lymphoma or “Hodgkin Lymphoma” or “Non-Hodgkin Lymphoma”). Reference lists of relevant articles were also screened manually for any additional possible records.

### Inclusion criteria

Studies included in this systematic review met the following criteria: (1) enrolled patients were diagnosed with paediatric lymphoma, including Hodgkin lymphoma and non-Hodgkin lymphoma; (2) studies investigated the diagnostic performance of FDG-PET/CT in the detection of BMI; (3) BMB was used as a (part of the) reference standard; and (4) sufficient data could be extracted to construct a 2 × 2 contingency table. Case reports, commentaries, expert opinion, narrative reviews, and studies carried out in animals and patients who had undergone systemic therapy were excluded. If more than one study provided overlapping data, only the most comprehensive or latest study was included.

### Data extraction

Requisite data extracted and recorded in standardized Excel files included surname of the first author, publication year, study inclusion interval, country, study design, demographic information of participants, number of BMI/total patients, interval between FDG-PET/CT and BMB, time between FDG administration and scanning, FDG dose, image analysis, criteria for positivity, standard reference, and number of false/true-positive and false/true-negative cases.

### Quality assessment

The methodological quality of the included studies was appraised according to the Quality Assessment of Diagnostic Accuracy Studies (QUADAS)-2 tool, which consists of four key domains (i.e. patient selection, index test, reference standard, and flow and timing). Risk of bias was assessed in each domain, and concerns about applicability were assessed in the first three domains with signalling questions. These questions were answered with “yes” for a low risk of bias/concern, “no” for a high risk of bias/concern, or “unclear” when relevant information was not clearly provided [9].

### Statistical analyses

Pooled sensitivity, specificity, positive likelihood ratio (PLR), negative likelihood ratio (NLR), and diagnostic odds ratio (DOR) were calculated using the bivariate meta-analysis framework. In addition, summarized receiver operating characteristic (sROC) curves were constructed, with the area under the curve (AUC) depicting the accuracy of the tests. Heterogeneity among the included studies was assessed using the I^2^ statistic. An I^2^ value of 0% implied no observed heterogeneity, and values of > 50% indicated substantial heterogeneity. For studies with substantial heterogeneity, we performed meta-regression analyses using a bivariate model to find the source of variability.

A two-sided *p* value < 0.05 was considered statistically significant in all statistical tests. Stata version 14 (StataCorp, College Station, TX) was used to analyse data from the included studies, and Review Manager Software version 5.3 (Cochrane Collaboration, Oxford, UK) was used to assess the methodological quality of the included studies.

## Results

### Search results and study selection

**Fig. 1:**
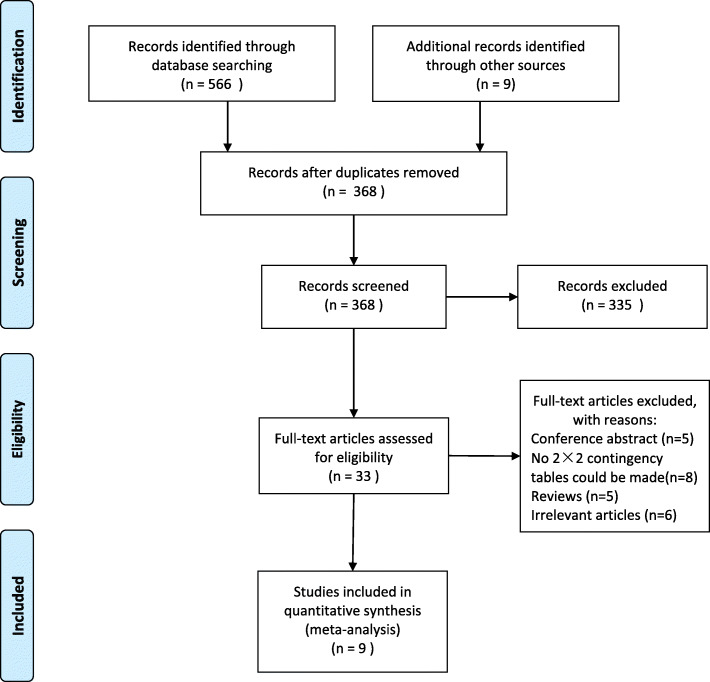
Selection process of included studies

A total of 1985 records were identified by searching databases and removing duplicates. After initial screening of titles and abstracts, 36 articles were further assessed by scrutinizing the full texts against the predesigned criteria, and 9 articles [11–19] were eventually included in the quantitative analysis. The selection processes for the eligible studies are depicted in Fig. 1.

### Study characteristics

One study [18] was prospective, eight studies [11–17, 19] were retrospective, and all the studies were cohort studies. Nine studies involving a total of 1640 patients (326 patients with BMI) explored the diagnostic accuracy of FDG-PET/CT for BMI in paediatric lymphoma patients; four of these studies [12, 16–18] were about Hodgkin’s lymphoma (HL), one study [14] was about non-Hodgkin’s lymphoma (NHL), and four studies [11, 13, 15, 19] were about both HL and NHL. The mean ages of the included patients ranged from 4 to 14.8 years, and the proportion of males ranged from 48.1 to 83.3%. The time between FDG administration and scanning ranged from 45 to 90 min, and the interval between FDG-PET/CT and BMB was within 14 days. The main characteristics of the included studies are summarized in Table 1.

**Table 1:** Characteristics of the included studies

Study (Published year)	Inclusion interval	Country	No. of patients	Male/Female	Mean Age (y) (range)	Study design/type	Time between FDG administration and scanning(min)
Yaǧci-Küpeli et al. [10]	2014.07-2014.12	Turkey	63	43/20	8.7 (2-17)	RCohort	60
Zapata et al. [11]	2009.01-2014.10	USA	69	32/37	9.6 (0.5-21)	RCohort	60
Cistaro et al. [12]	NR	Italy	224	NR	14 (4-18)	RCohort	60-90
Chen et al. [13]	2010.05-2017.02	China	93	66/27	8 (1-21)	RCohort	60
Badr et al. [14]	2010.02-2015.12	Egypt	140	105/35	8.6 (2-17)	RCohort	45-60
Hassan et al. [15]	2010.07-2015.06	Pakistan	784	653/131	10.3 (2-18)	RCohort	60
Agrawal et al. [16]	NR	India	38	30/8	9.8 (3-18)	RCohort	45-60
Purz et al. [17]	2002-2006	Germany	175	89/86	14.6	PCohort	40-90
Cheng et al. [18]	2007.07-2008.12	USA	54	26/28	14.8 (6-24)	RCohort	60-90
Study (Published year)	Interval between FDG-PET/CT and BMB	FDG dose	Criteria for positivity	Reference standard		Lymphoma type	
Yaǧci-Küpeli et al, [10]	NR	185 MBq	Bone marrow uptake was higher than the liver	BMB; PET/CT; follow-up		HL and NH	
Zapata et al, [11]	NR	NR	FDG avidity was equal to primary tumor or greater than adjacent tissues	BMB; PET/CT; follow-up		HL and NHL	
Cistaro et al, [12]	<15 days	weight-adapted^a^	isolated/multiple focal uptake was higher than the liver or spleen^b^	BMB; PET/CT; follow-up		HL	
Chen et al, [13]	1-14 days	5.18 MBq/Kg	Focal or multifocal abnormally increased FDG uptake	BMB; PET/CT; follow-up; MRI		NHL	
Badr et al, [14]	<14 days	5 to 10 MBq/kg,	Bone marrow uptake was higher than the liver	BMB; PET/CT; followup; MRI		HL and NHL	
Hassan et al, [15]	<14 days	weight-based (90–270 MBq)	one or more bifocal uptake^b^	BMB; PET/CT; follow-up		HL	
Agrawal et al, [16]	NR	3.7 MBq/kg	focal uptake was higher than the liver^b^	BMB; PET/CT; follow-up		HL	
Purz et al, [17]	NR	weight–adapted	isolated/multiple focal uptake was higher than the liver	BMB; PET/CT; follow-up		HL	
Cheng et al, [18]	NR	5.18 MBq/kg or 0.14 mCi/kg	focal or multifocal abnormally increased FDG uptake	BMB; PET/CT; follow-up		HL and NHL	

*P* prospective, *R* retrospective, *NR* not reported, *BMB* bone marrow biopsy, *CT* computed tomography, *FDG* 18F-fluoro-2-deoxy-D-glucose, *PET* positron emission tomography, *BMI* bone marrow involvement, *MRI* magnetic resonance imaging

^a^ Weight-adapted FDG dosage recommended according to the manufacturer guidelines for each scan model

^b^ Diffusely/homogeneously increased bone marrow FDG uptake was also regarded as positive for BMI

### Results of quality assessment

**Fig. 2:**
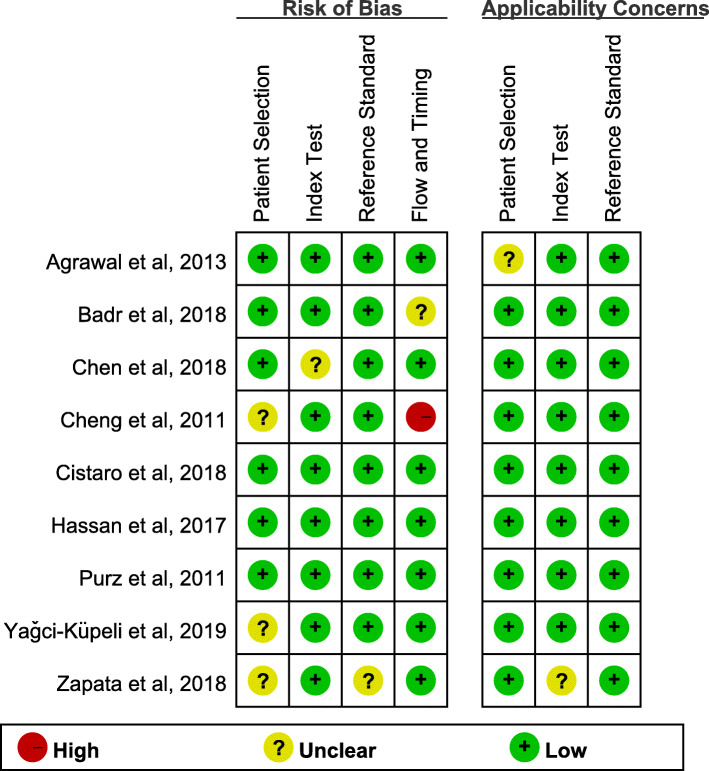
Quality assessment of included studies using QUADAS-2 tool criteria. Red in figure indicates high risk, yellow represents unclear risk and green means low risk

The results of the QUADAS-2 assessments for each included study are displayed in Fig. 2. In almost every key domain, the proportion of high-risk and unclear studies was less than 20%, which indicated that the quality of the included studies was good.

### Diagnostic performance

**Fig. 3:**
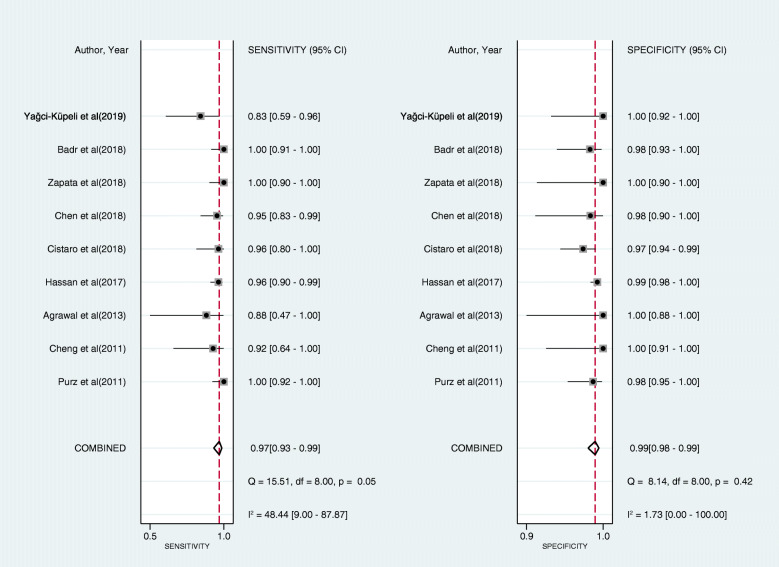
Forest plots of the sensitivity and specificity of FDG-PET/CT for bone marrow infiltration in the newly diagnosed paediatric lymphoma across all included studies. Diamonds in the central vertical lines represent pooled sensitivities or specificities with corresponding 95% confidence interval

**Fig. 4:**
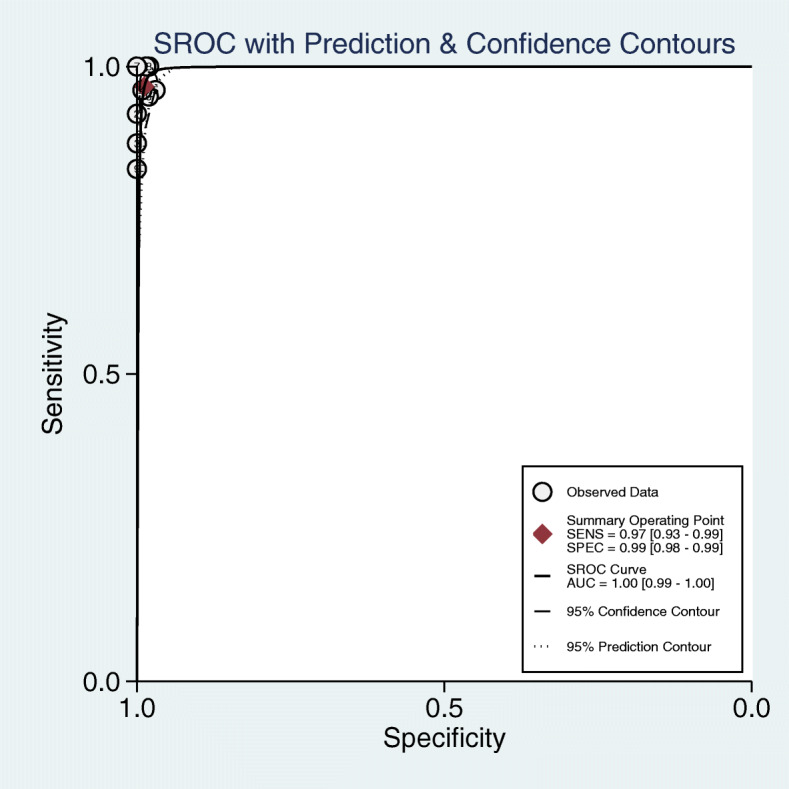
Summarized receiver operating characteristic curve (sROC) of FDG-PET/CT for bone marrow infiltration in the newly diagnosed paediatric lymphoma with corresponding 95% confidence region and the 95% prediction region

**Fig. 5:**
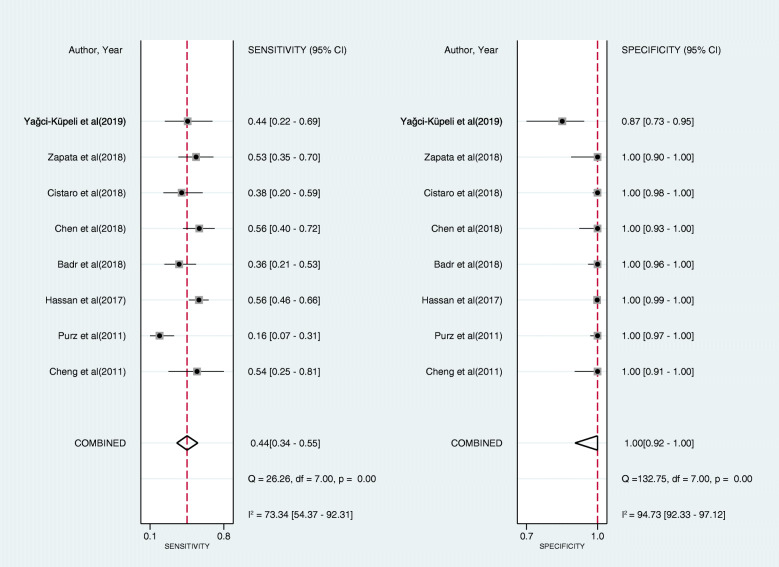
Forest plots of the sensitivity and specificity of bone marrow biopsy for bone marrow infiltration in the newly diagnosed paediatric lymphoma across all included studies. Diamonds in the central vertical lines represent pooled sensitivities or specificities with corresponding 95% confidence interval

As shown in Fig. 3, the pooled sensitivity and specificity of FDG-PET/CT for diagnosing BMI in newly diagnosed paediatric lymphoma patients were 0.97 (95% CI, 0.93 to 0.99) and 0.99 (95% CI, 0.98 to 0.99), respectively. The pooled PLR, NLR, and DOR were 79.9 (95% CI, 42.7 to 149.6), 0.03 (95% CI, 0.01 to 0.17), and 2414.6 (95% CI, 989.6 to 5891.4), respectively. The AUC of FDG-PET/CT for BMI was 1.00 (95% CI, 0.99 to 1.00) (Fig. 4). The I^2^ statistics for sensitivity and specificity values were 48.44% (95% CI, 9.00% to 87.87%, *p* value = 0.05) and 1.73% (95% CI, 0.00% to 100.00%, *p* value = 0.42), respectively, which indicated no substantial heterogeneity among the included studies. Compared with PTE/CT, BMB had a lower pooled sensitivity (0.44, 95% CI, 0.34 to 0.55) and comparable pooled specificity (1.00, 95% CI, 0.92 to 1.00) (Fig. 5). The summary results of bivariate model analysis and subgroup analysis are presented in Table 2.

**Table 2:** Summary results of bivariate model analysis and sub-group analysis

Summary results of bivariate model analysis						
Bivariate Model Analysis						
Sen (95% CI)	Spe (95% CI)	PLR (95% CI)	NLR (95% CI)	DOR (95% CI)	AUC (95% CI)	
PET/CT						
0.97(0.93-0.99)	0.99(0.98-0.99)	79.9(42.7-149.6)	0.03(0.01-0.17)	2414.6(989.6-5891.4)	1.00(0.99-1.00)	
BMB						
0.44(0.34-0.55)	1.00(0.92-1.00)	1277.1(4.8-337317.5)	0.56(0.46-0.68)	2274(9-596153)	0.71(0.67-0.75)	
Subgroup Analyses						
Subgroup	Sen (95% CI)	Spe (95% CI)	PLR (95% CI)	NLR (95% CI)	DOR (95% CI)	AUC (95% CI)
PET/CT						
HL	0.97(0.93-0.99)	0.99(0.97-0.99)	66.4(36.7-120.3)	0.03(0.01-0.07)	2532(865-7412)	1.00(0.99-1.00)
NHL	0.94(0.85-0.97)	0.99(0.94-1.00)	68.9(15.7-302.9)	0.06(0.03-0.16)	1062(193-5853)	0.99(0.98-1.00)
BMB						
HL	0.32(0.18-0.50)	1.00(0.91-1.00)	231.8(2.9-18534.3)	0.68(0.53-0.87)	341(4-30241)	0.65(0.61-0.69)
NHL	0.55(0.45-0.64)	0.99(0.95-1.00)	77.1(10.8-547.6)	0.46(0.37-0.56)	169(23-1250)	0.98(0.96-0.99)

### Publication bias

**Fig. 6:**
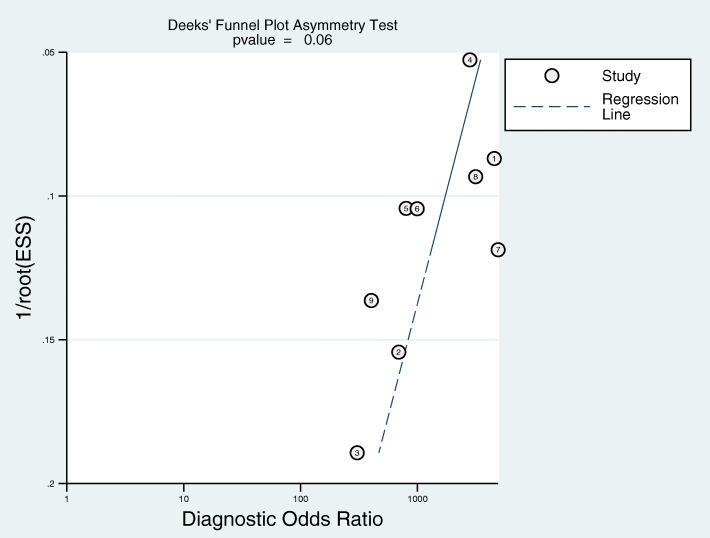
Deek’s funnel plot asymmetry test indicated no evidence of significant publication bias

Deek’s funnel plot asymmetry test indicated no evidence of significant publication bias (*p* = 0.06) (Fig. 6).

## Discussion

Accurate and fast diagnosis of BMI in newly diagnosed lymphoma patients remains a challenging problem. The present staging and response criteria for HL and NHL published by the National Cancer Institute Working Group and revised by the International Working Group (IWG) in 2007 have been adopted by most physicians [20]. BMB remains the most commonly used method and gold standard for the clinical assessment of BMI. Despite the guidelines issued by the European Society for Medical Oncology (ESMO) in 2011 and 2018 indicating that FDG-PET/CT is sufficient for BMI assessment in adults, the effectiveness of FDG-PET/CT in evaluating paediatric lymphoma is still ambiguous since relevant data are scarce [20]. Our results revealed that FDG-PET/CT was highly sensitive and specific in the BMI evaluation of newly diagnosed paediatric lymphoma, with a pooled sensitivity of 0.97 (95% CI, 0.93~0.99) and pooled specificity of 0.99 (95% CI, 0.98~0.99).

As a systemic metabolic imaging technique that has been widely used in metastatic and invasive malignancies, FDG-PET/CT has been considered to have the potential for evaluating BMI in lymphoma patients. In 2010, Riad et al. [20] compared the role of FDG-PET/CT versus CT in the evaluation of paediatric lymphoma at the initial, intermediate chemotherapy, end of treatment, and recurrence stages based on 152 patients. In the initial staging, FDG-PET/CT staging was more accurate than CT in 11 of 41 patients. They demonstrated that FDG-PET/CT is of great significance in the early evaluation of paediatric lymphoma. The results of the present meta-analysis showed that the pooled specificity of FDG-PET/CT and BMB in the diagnosis of BMI in newly diagnosed paediatric lymphoma patients was approximate, but the pooled sensitivity of FDG-PET/CT was significantly higher than that of BMB. This suggests that FDG-PET/CT may be an option to avoid BMB in children with newly diagnosed lymphoma. In 2013, Adams et al. [21] found that in evaluating BMI in lymphoma, FDG-PET/CT could detect BMI patients who were not detected by BMB. This indicates that FDG-PET/CT can accurately detect BMI in lymphoma patients.

Previous studies have confirmed that FDG-PET/CT has a high sensitivity and specificity for evaluating BMI in HL patients, while its effectiveness for evaluating BMI in NHL patients has not been confirmed [21]. Our subgroup analysis found that the pooled sensitivity (0.97 and 0.94, respectively) and specificity (0.99 and 0.99, respectively) of FDG-PET/CT for evaluating BMI in HL and NHL patients were both high. In addition, the sensitivity of FDG-PET/CT-assessed HL and NHL was significantly higher than that of BMB (0.32 and 0.55, respectively). In addition, although the sensitivity of FDG-PET/CT for evaluating BMI in NHL patients was slightly lower than that of HL, there was no difference in specificity. The heterogeneity and publication bias of this study were very small; in fact, after removing the study by Yaǧci-Küpeli et al. [10], both the heterogeneity and publication bias vanished. The *I*^*2*^ of sensitivity and specificity were 48.44% and 1.73%, and the *P* values were 0.05 and 0.42, respectively. After removing the study by Yaǧci-Küpeli et al. [10], the *I*^*2*^ of sensitivity and specificity became 13.21% and 3.79%, and the *P* value changed to 0.33 and 0.40, respectively. There was no threshold effect for evaluating BMI in lymphoma patients using FDG-PET/CT or BMB, so no further threshold effect analysis was performed.

BMB is an invasive test that causes pain to the patient [22] and may cause some complications that cannot be ignored [23]. Studies have shown that BMB assesses bone marrow involvement by detecting only a small fraction of the bone marrow and is therefore prone to sampling errors [24]. For newly diagnosed paediatric lymphoma patients, false-negative BMI is more likely to be assessed using BMB. In contrast, FDG-PET/CT is a non-invasive systemic metabolic imaging technique that visualizes the entire bone marrow and has the advantages of non-invasive and error-free sampling over BMB [21]. Given all the paediatric lymphomas that are metabolically FDG-avid, FDG-PET/CT is valuable for the evaluation of lymphoma infiltration, especially BMI. The diagnosis of BMI is critical in assessing the disease status of lymphoma, guiding Ann Arbor staging, and influencing prognosis and treatment. Adequate examination before treatment can reduce the risk of litigation [10, 25]. In addition, the accuracy of FDG-PET/CT in BMI diagnosis is better than that of BMB, so FDG-PET/CT should be used instead of BMB.

The strengths of the current study lie in the following two aspects. First, we innovatively demonstrated the efficacy of FDG-PET/CT in evaluating the BMI of newly diagnosed paediatric malignant lymphoma. Second, a subgroup analysis was conducted; thus, we demonstrated that FDG-PET/CT is effective in assessing BMI in NHL patients, which is unprecedented. Potential limitations of this meta-analysis should also be considered. The included studies were mainly retrospective studies, with only one prospective study lacking prospective confirmatory studies. Research on FDG-PET/CT assessment of inert lymphoma is still insufficient, and further studies are needed to obtain more data in the future.

## Conclusions

Based on the results of the current meta-analysis, it could be concluded that compared with BMB, FDG-PET/CT was a more valuable diagnostic method for evaluating BMI in paediatric Hodgkin and non-Hodgkin lymphoma patients with extremely high diagnostic accuracy.

## Data Availability

The datasets used and/or analysed during the present study are available from the corresponding author on reasonable request.
